# Abnormal expression and processing of the proprotein convertases PC1 and PC2 in human colorectal liver metastases

**DOI:** 10.1186/1471-2407-5-149

**Published:** 2005-11-17

**Authors:** George N Tzimas, Eric Chevet, Sarah Jenna, Duc Thang Nguyên, Abdel M Khatib, Victoria Marcus, Yi Zhang, Michel Chrétien, Nabil Seidah, Peter Metrakos

**Affiliations:** 1 Transplant and Hepato-pancreatobiliary Research Group, McGill University Health Center, McGill University, 687 Pine Avenue West, S.10.26, Montreal, Canada H3A1A1; 2 Department of Surgical Services, Kypselis General Hospital, 24 Drossopoulou Street, Athens 11257, Greece; 3 Organelle Signaling Laboratory, Department of Surgery, McGill University Health Center, McGill University, 687 Pine Avenue West, S.10.26, Montreal, Canada H3A1A1; 4 Ottawa Health Research Institute, University of Ottawa, Ottawa, Canada Y1K 4K9; 5 Department of Pathology, McGill University Health Center, McGill University, 1650 Cedar Avenue, Montreal, Canada H3G 1A4; 6 Laboratory of Biochemical Neuroendocrinology, Clinical Research Institute of Montreal, University of Montreal, Montreal, Canada H2W 1R7, QC

## Abstract

**Background:**

The family of proprotein convertases has been recently implicated in tumorigenesis and metastasis in animal models. However, these studies have not yet been completely corroborated in human tumors.

**Methods:**

Using RT PCR, immunoblot and immunohistochemistry we assessed the presence and the processing patterns of the convertases PC1 and PC2 as well as the PC2 specific chaperone 7B2 in human liver metastases originating from colorectal cancer and compared them to unaffected and normal liver. Furthermore, we assessed the presence and processing profiles of PC1, PC2 and 7B2 in primary colon cancers.

**Results:**

mRNA, protein expression, and protein cleavage profiles of proprotein convertases 1 and 2 are altered in liver colorectal metastasis, compared to unaffected and normal liver. Active PC1 protein is overexpressed in tumor, correlating with its mRNA profile. Moreover, the enhanced PC2 processing pattern in tumor correlates with the overexpression of its specific binding protein 7B2. These results were corroborated by immunohistochemistry. The specific and uniform convertase pattern observed in the metastases was present only in a fraction of primary colon cancers.

**Conclusion:**

The uniformly altered proprotein convertase profile in liver metastases is observed only in a fraction of primary colon cancers, suggesting possible selection processes involving PCs during metastasis as well as an active role of PCs in liver metastasis. In addition, the exclusive presence of 7B2 in metastatic tumors may represent a new target for early diagnosis, prognosis and/or treatment.

## Background

Liver metastasis remains the major cause of therapeutic failure in colorectal carcinoma. Amongst the 300,000 people that are given the diagnosis of colorectal cancer (CRC) every year in Europe and North America, 60% will eventually develop liver metastases, and several of them will succumb from their disease. Metastasis microenvironment conditioned by the tumor itself, may represent an alternative to the "seed-soil" theory. Indeed recent reports have demonstrated the pivotal role of the proprotein convertases (PCs) (1) in tumor growth and metastasis of HT-29 human colon carcinoma cells (2), lung (3) or breast cancer cells (4). To date eight members of this family have been identified, namely furin, PC1/PC3, PC2, PC4, PACE4, PC5/PC6, PC7/LPC/PC8 and SKI-1/S1P [1-7]. Amongst the PCs, PC1 and PC2 are unique due to their processing activity specific to the regulated secretory pathway, and their localization in dense core secretory granules. Both proteins are synthesized in the Endoplasmic Reticulum (ER) as zymogens (primary cleavage of the zymogen of proPC1 occurs in the ER while that of proPC2 occurs in the granules), transported through the Golgi apparatus, and the Trans Golgi Network (TGN) into mature secretory granules, and both are proteolytically activated along the way. The proper trafficking and processing of proPC2 into its active form is dependent on 7B2, a chaperone-like protein [8]. This bifunctional polypeptide has been shown to promote proPC2 cleavage to the active PC2 form with its N-terminal domain, but also to inhibit further PC2 processing with its C-terminal domain. The exact biological significance of this protein has not been elucidated yet, although its deletion in mice leads to a Cushing's-like syndrome [9]. Finally, recently a specific PC1 inhibitor, ProSAAS has been identified [10], but its physiological function remains unclear.

The purpose of this study is to examine the expression and processing patterns of PC1 and PC2 in human CRC liver metastases and to compare these profiles with those observed in unaffected and normal liver. Furthermore, we assessed the processing patterns of these convertases in primary colon cancers. Finally, we attempt to shed light into the PC1 and PC2 processing mechanisms and their potential impact on CRC liver metastasis.

## Methods

### Human liver specimens

Tumor specimens and their unaffected counterparts (Fig. 7D) were collected from 14 patients (proper informed consent obtained) that underwent liver resection for CRC metastases. Normal liver specimens were obtained from organ donors. All the specimens were harvested immediately after resection and were snap frozen in liquid nitrogen.

### Human colon cancer specimens

In a similar fashion, colon cancer specimens with unaffected colon were collected from 5 patients (proper informed consent obtained) that underwent colon resection for primary colon cancer (pathology proven adenocarcinoma). The specimens were harvested immediately after resection and were snap frozen in liquid nitrogen.

### PCR primers

PC1 primers were designed as follows f5'-TGGCTTGCTAAATGCCAAAGCTC-3'/r5'-ATCCACCATCTTCTCCACCCC-3' (annealing temperature 60°C), PC2 primers were f5'-GTCCTTGATGCAGGTGCCATC-3'/r5'-ACTCCTTCAGCACCCCCTTC-3' (annealing temperature 62°C) and 7B2 primers were f5'-CAC CAG GCC ATG AAT CTT-3'/r5'-CTG GAT CCT TAT CCT CAT CTG-3' (annealing temperature 61°C. GAPDH was used as standard and for normalization (f5'-CCC TTC ATT GAC CTC AAC TAC ATG GT-3'/r5'-GAG GGG CCA TCC ACA GTC TTC TG-3') (annealing temperature 58°C).

### Antibodies

**Figure 1:**
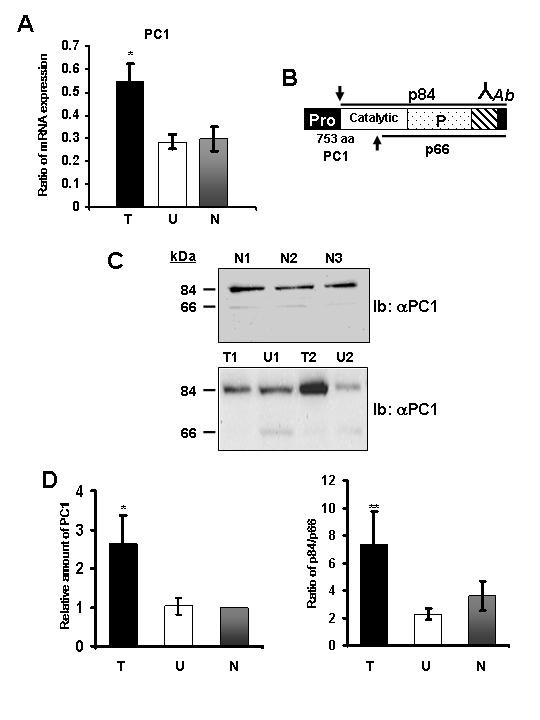
**Expression of PC1 in normal and unaffected liver compared to colorectal (CRC) liver metastases**. **A**. Quantification of PC1 mRNA expression respectively, in 3 normal (N), 14 tumor (T) and 14 unaffected (U) liver samples. Quantification was obtained by the ratio of the optical density of PC1 PCR amplification products over GAPDH. Standard error of mean (SEM) is indicated. Asterisk indicates a statistically significant difference (*P *< 0.04). **B. **Schematic representation of PC1 structure. The antigenic region against which the antibody was raised is mentioned (YAb). Cleavage sites are indicated (arrows). **C. **PC1 cleavage profile in normal (N1-N3, top panel), unaffected (U1-U2) and tumor (T1-T2, bottom panel) samples. Normal liver samples (N1-N3) derived from three different organ donors. T1/U1 and T2/U2 came from two independent patients. PC1 immunoblotting was done on a total of 3 normal, 14 metastasis and 14 unaffected liver samples, and was repeated three times. **D. **Relative amounts of total PC1 (C, left panel) protein, in tumor (T), unaffected (U) and normal liver (N), expressed as a ratio over normal liver samples. Ratios of p84/p66 PC1 isoforms (C, right panel) in the same samples are calculated. SEM is indicated. Simple and double asterisks indicate statistically significant differences (P < 0.05).

**Figure 2:**
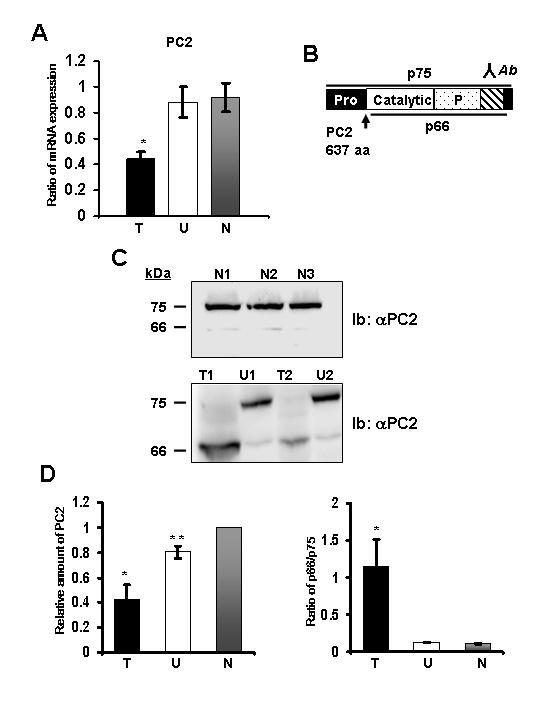
**Expression of PC2 in normal and unaffected liver compared to colorectal (CRC) liver metastases**. **A**. Quantification of PC2 mRNA expression respectively, in 3 normal (N), 14 tumor (T) and 14 unaffected (U) liver samples. Quantification was obtained by the ratio of the optical density of PC2 PCR amplification products over GAPDH. Standard error of mean (SEM) is indicated. Asterisk indicates a statistically significant difference (*P *< 0.003). **B. **Schematic representation of PC2 structure. The antigenic region against which the antibody was raised is mentioned (YAb). Cleavage sites are indicated (arrows). **C. **PC2 cleavage profile in normal (N1-N3, top panel), unaffected (U1-U2) and tumor (T1-T2, bottom panel) samples. Normal liver samples (N1-N3) derived from three different organ donors. T1/U1 and T2/U2 came from two independent patients. PC1 immunoblotting was done on a total of 3 normal, 14 metastasis and 14 unaffected liver samples, and was repeated three times. **D. **Relative amounts of total PC2 (C, left panel) protein, in tumor (T), unaffected (U) and normal liver (N), expressed as a ratio over normal liver samples. Ratios of p75/p66 PC2 isoforms (C, right panel) in the same samples are calculated. SEM is indicated. Simple and double asterisks indicate statistically significant differences (P < 0.05).

**Figure 3:**
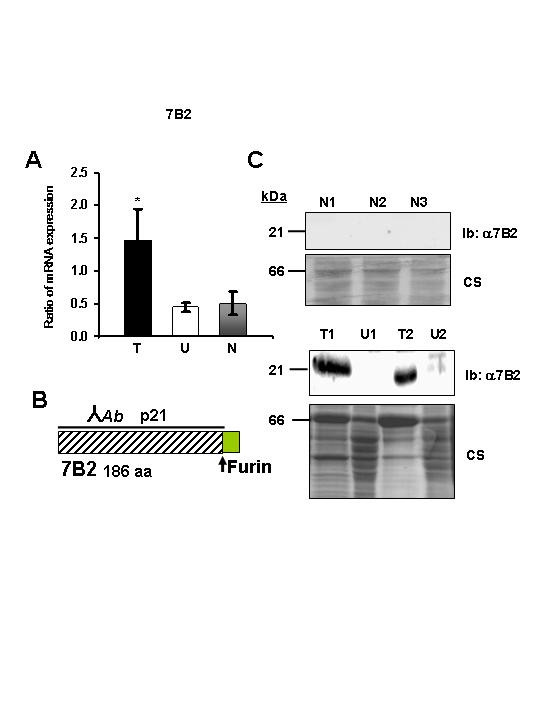
**Expression of 7B2 in normal and unaffected liver compared to colorectal (CRC) liver metastases**. **A**. Quantification of 7B2 mRNA expression respectively, in 3 normal (N), 14 tumor (T) and 14 unaffected (U) liver samples. Quantification was obtained by the ratio of the optical density of 7B2 PCR amplification products over GAPDH. Standard error of mean (SEM) is indicated. Asterisk indicates a statistically significant difference (*P *< 0.003). **B. **Schematic representation of 7B2 structure. The furin cleavage site is shown (arrow). The antigenic region against which the antibody was raised is indicated (YAb). **C. **7B2 immunoblots in normal liver (N1-N3, top panel), in tumor (T1, T2, bottom panel) and unaffected (U1, U2, bottom panel) liver, indicating presence of 7B2 protein only in tumor. Corresponding gels stained with Coomassie blue G250 (CS) are shown. Experiments were repeated three times.

Rabbit antisera raised either against PC1 (amino acids 621–726, Figure 1B), PC2 (amino acids 529–637, Figure 2B) or 7B2 (amino acids 32–39, Figure 3B) were used.

### RNAs and proteins extraction

RNA was extracted using Trizol (Invitrogen) according to the manufacturer's instructions. Semi-quantitative RT-PCR analysis was performed as previously described [11]. For protein extraction, approximately 0.5 mg of sample tissues were homogenized as previously described [12]. TX-100 was added to a 1% final concentration and tissue extract solubilized for 30' at 4°C. Lysates were then centrifuged at 10000 g for 2 × 30' at 4°C. Protein concentrations in the supernatants were determined using the Bradford method. For every immunoblot analysis 100 μg of protein were used.

### Immunohistochemistry

4 μm-thick, formalin-fixed, paraffin-embedded tissue sections, were treated as previously described [13]. The slides were incubated with anti-PC1, PC2 and 7B2 antibodies at a 1:2000 dilution, revealed with biotinylated goat-anti-rabbit IgG-B (1:200), combined with Histocain-plus kit, followed by 3-amino-9-ethylcarbazole (AEC) chromogen and counterstaining with hematoxylin.

### Statistical analysis

Data are expressed as means ± SD. Comparisons between the specimens were performed using student's t-test. A value of p < 0.005 was considered statistically significant.

## Results

### PC1 and PC2 mRNA are expressed in both normal and unaffected liver but their expression is differentially regulated in tumor

We initially assessed the presence of the mRNA encoding PC1 and PC2 in liver CRC metastatic tumors versus unaffected and normal livers. PC1 and PC2 RT-PCR amplification products were of the expected size, 553 bp and 422 bp respectively. PC1 mRNA expression was two-fold higher in areas of tumor compared to areas of unaffected and normal liver (P < 0.04, Figure 1A). In contrast, PC2 mRNA was overexpressed two-fold in areas of unaffected and normal liver compared to areas of metastasis (P < 0.003, Figure 2A).

### PC1 and PC2 protein expression and maturation are altered in tumor

To correlate mRNA and protein expression, we examined total sample lysates by immunoblotting. Relative amounts of protein were quantified by scanning densitometry of the immunoblots. Interestingly, the anti-PC1 antibody (Fig. 1B) revealed two immunoreactive species of 84 and 66 kDa, respectively (Fig. 1C), in tumor (T), unaffected (U), and normal (N) liver. The higher molecular mass isoform (84 kDa) corresponded to the full-length active PC1 whereas the C-terminally immunoreactive 66 kDa isoform was likely to be an N-terminally truncated, inactive form of PC1 (Fig. 1B) (14,15). In tumors the total amount of PC1 (p84+p66) was ~2.5-fold more elevated than in unaffected and normal samples (Fig. 1D, left panel). Moreover the ratio of the 84 kDa active form over the 66 kDa form was also ~2.5 times higher than in both unaffected and normal liver (Fig. 1D, right panel). Immunoblot analysis with anti-PC2 antibody (Fig. 2B) revealed two species of 75 and 66 kDa respectively, representing the inactive proPC2 isoform and the cleaved, active PC2 (Fig. 2C) [14]. In contrast to PC1, the amount of total PC2 (p75+p66) was higher in unaffected and normal liver than in metastasis (Fig. 2D, left panel). Nevertheless a similar pattern of processing was observed, i.e. in metastasis the fully active 66 kDa form predominates over the inactive 75 kDa pro-form, while in unaffected and normal liver the proPC2 isoform was more abundant. Again the PC2 processing ratio of active PC2 (p66) over the inactive proPC2 (p75) in tumor versus unaffected and normal liver indicated a ~10-fold increase in PC2 maturation in liver metastasis (Fig. 2D, right panel). In conclusion, alteration of PC1 and PC2 processing, lead to increased accumulation of active isoforms of both convertases in liver metastasis.

### The PC2 chaperone, 7B2, is expressed only in liver CRC metastases

Since PC2 processing is controlled by its specific binding protein 7B2, we quantified 7B2 expression profiles in normal and unaffected livers compared to liver metastasis. RT-PCR amplification of 7B2 led to an expected 454 bp product and showed a ~3-fold (P < 0.003) increase of 7B2 mRNA in metastasis compared to unaffected or normal liver (Fig. 3A). By immunoblotting, we were not able to detect any 7B2 in both unaffected and normal liver, while an intense immunoreactive band was observed at the expected 7B2 molecular mass of 21 kDa [8]) in metastasis samples (Fig. 3C).

### Immunohistochemical validation

**Figure 4:**
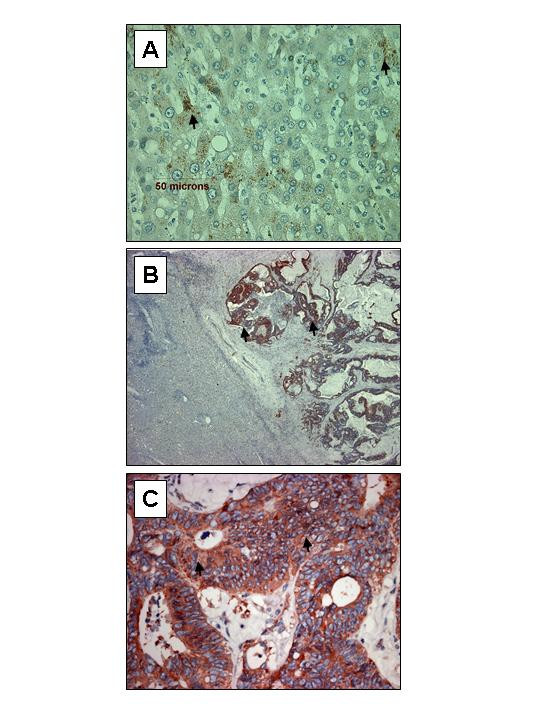
**Immunohistochemistry for PC1 in normal and unaffected liver compared to colorectal (CRC) liver metastases**. **A. **Light microscopy immunohistochemistry of normal liver (N), using 100 × magnification. Arrowheads indicate the scarcely positively stained hepatic cells. **B. **Light microscopy PC1 immunohistochemistry of liver metastasis (T) and adjacent unaffected parenchyma (U), using 20 × magnification. Arrowheads indicate positively stained tumor cells. **C. **Light microscopy PC1 immunohistochemistry of liver metastasis (T) using 400 × magnification. Arrowheads indicate positively stained tumor cells.

**Figure 5:**
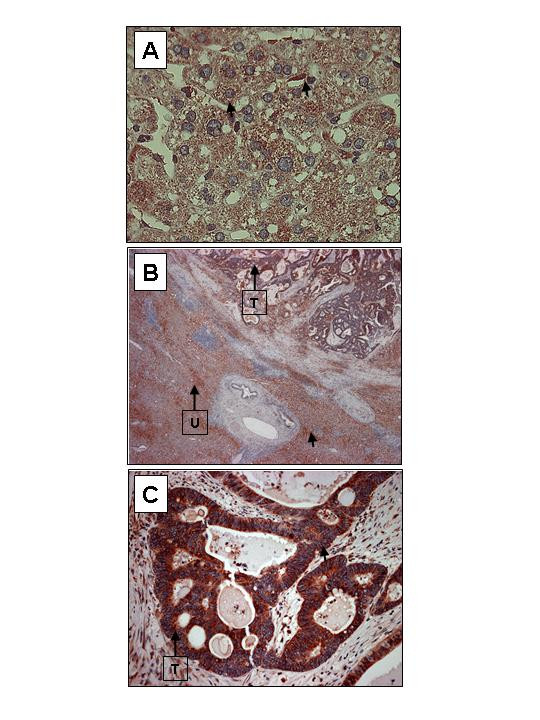
**Immunohistochemistry for PC2 in normal and unaffected liver compared to colorectal (CRC) liver metastases**. **A. **Light microscopy PC2 immunohistochemistry of normal liver (N), using 400 × magnification. Arrowheads indicate positively stained hepatic cells. **B. **Light microscopy PC2 immunohistochemistry of liver metastasis (T) and adjacent unaffected parenchyma (U), using 25 × magnification. Arrowheads indicate positively stained cells mainly in the unaffected liver. **C. **Light microscopy PC2 immunohistochemistry of liver metastasis (T) using 200 × magnification. Arrowheads indicate positively stained tumor cells.

**Figure 6:**
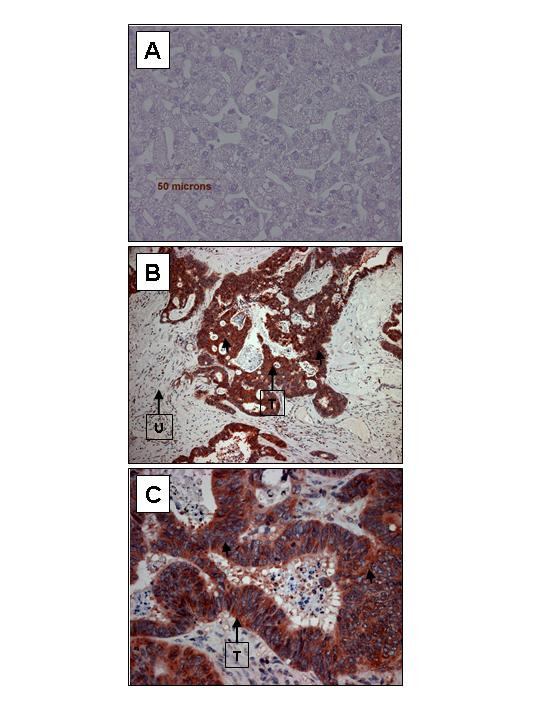
**Immunohistochemistry for 7B2 in normal and unaffected liver compared to colorectal (CRC) liver metastases**. **A. **Light microscopy 7B2 immunohistochemistry of normal liver (N), using 400 × magnification. No positive cells were identified. **B. **Light microscopy 7B2 immunohistochemistry of liver metastasis (T) and adjacent unaffected parenchyma (U), using 100 × magnification. Arrowheads indicate positively stained cells uniquely in the metastasis. **C. **Light microscopy 7B2 immunohistochemistry of liver metastasis (T) using 400 × magnification. Arrowheads indicate dense positively stained tumor cells.

Using the antibodies described above, we characterized PC1, PC2 and 7B2 localization by immunohistochemistry. PC1 staining was cytoplasmic and more abundant in areas of tumor than in adjacent unaffected liver, where only a few cells were positively stained (Fig. 4B, 4C), thus correlating with the mRNA and immunoblot data. PC2 was present in areas of tumor, and adjacent unaffected parenchyma (Fig. 5B, 5C). In addition, 7B2 was detected by very strong cytoplasmic staining in areas of liver metastasis while adjacent unaffected liver did not stain (Fig. 6B, 6C). Finally, although normal liver stained for PC1 (Fig. 4A) and PC2 (Fig. 5A), 7B2 remained undetectable (Fig. 6A).

### The expression and processing pattern of PC1 and PC2 observed in metastases is only found in a subset of primary colon cancers

**Figure 7:**
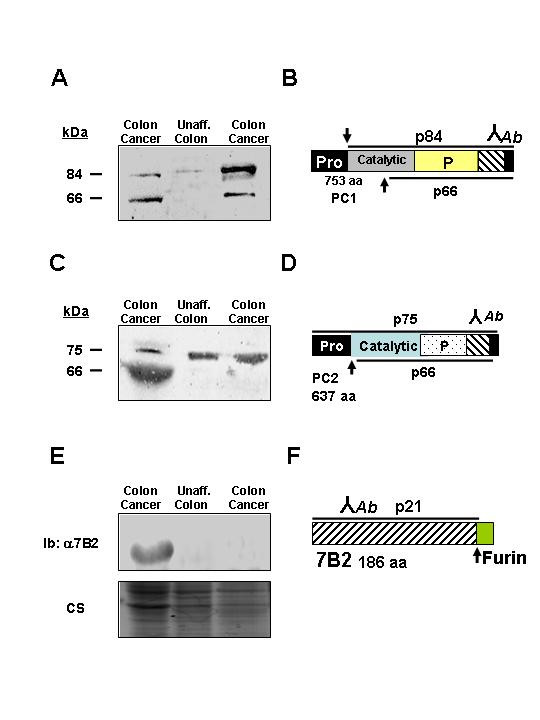
**Expression of PC1, PC2 and 7B2 in primary colon cancer and unaffected colon**. **A. **PC1 immunoblots in primary colon cancer in unaffected colon and in an additional primary colon cancer, note the different PC1 processing pattern between the two colon cancers. Experiments were repeated three times. **B. **Schematic representation of PC1 structure. **C. **PC2 immunoblots in primary colon cancer in unaffected colon and in an additional primary colon cancer. Note the absence of the 66 Kda band in the second primary colon cancer. Experiments were repeated three times. **D. **Schematic representation of PC2 structure. **E. **7B2 immunoblots in primary colon cancer in unaffected colon and in an additional primary colon cancer, indicating presence of 7B2 protein only in one tumor. Experiments were repeated three times. Corresponding gels stained with Coomassie blue G250 (CS) are shown. **F. **Schematic representation of 7B2 structure.

Using the antibodies described above, we assessed the presence as well as the processing profile of PC1 and PC2 in primary colon cancers using immunoblot. PC1 was detected in various amounts in primary colon cancers but was undetectable in their unaffected counterparts (Fig. 7A). Immunoblot analysis revealed two immunoreactive species of 84 and 66 kDa, respectively. The higher molecular mass isoform (84 kDa) corresponded to the full-length active PC1 whereas the C-terminally immunoreactive 66 kDa isoform was likely to be an N-terminally truncated, inactive form of PC1. The ratio of the two isoforms varied among specimens with 3 out of 5 specimens expressing more abundant the 84 kDa isoform. PC2 was also detected in various amounts in primary colon cancers and was undetectable in their unaffected counterparts (Fig. 7B). Furthermore, the active 66 kD isoform was more abundant in 2 out of 5 specimens. In these specific specimens (2 out of five), immunoblot analysis revealed an intense immunoreactive band at the expected 7B2 molecular mass of 21 kDa (Fig. 7C). These results demonstrate a strong heterogeneity in the expression/processing of PC1, PC2 and 7B2 in primary colon cancers compared to liver metastases, As a consequence they suggest that the expression/processing pattern of these convertases observed in liver metastases may represent an important feature of these cancers.

## Discussion

In this study, we assessed the presence and the cleavage/processing patterns of the two major convertases of the regulated secretory pathway, PC1 and PC2, as well as the PC2 chaperone 7B2. This study was performed in human liver metastases specimens from colorectal primaries and in unaffected liver samples from the same patients that underwent liver resection, as well as in normal livers. We also assessed these convertase profiles in primary colon cancers.

To our knowledge this is the first report describing the expression of PC1, PC2 and 7B2 in human liver tissues, and in human colon cancers. More importantly, we noted that (i) at the mRNA level, PC1 is overexpressed in metastatic tumor versus unaffected and normal liver, while PC2 expression is downregulated (Fig. 1A and 2A respectively); (ii) consistently, at the protein level, PC1 (p84+p66) is overexpressed (Fig. 1D), while PC2 (p75+p66) is downregulated in metastatic tumor (Fig. 2D); (iii) both active PC1 and PC2 are predominant in metastatic tumor (Fig. 1D and 2D respectively); (iv) consistently with the enhanced PC2 zymogen processing pattern, in tumor, 7B2 is overexpressed (Fig. 3A and 3C); (v) the above results are corroborated by immunohistochemistry (Fig. 4, 5, 6).

We also found that the specific PC2 and 7B2 profile observed in metastatic cancers was observed only in a fraction of primary colon cancers. These data support a specific negative feedback mechanism regulating PC2 mRNA expression in liver metastases, when PC2 proteolytic activity is overwhelming. Therefore in metastatic tumors, abundant active PC2 may lead to a downregulation of its mRNA, and vice versa in unaffected and normal liver. Furthermore, the homogeneous liver metastasis PC2 protein profile supports the hypothesis of a tightly regulated active PC2 production in the primary as well as in metastatic tumors, with 7B2 playing a leading role in this mechanism. Interestingly, PC1, PC2 and 7B2 are considered as markers of endocrine and neuroendocrine phenotypes [5,8,12]. The fact that they are also detected in human anal canal [13] from which also cancers and eventually metastases can develop suggests that a neuroendocrine differentiation program could take place during colon carcinogenesis and liver metastasis. Recently, a specific RE1-lk silencer element in the promoter of PC2 was identified [17] and binding of transcription-silencing factors to this element may contribute to repression of the PC2 gene in non-neuroendocrine cells.

Our results are in agreement with recent experimental animal evidence highlighting the potential implication of convertases in tumorigenesis and metastasis. Indeed, Khatib *et al. *have shown that convertase inhibition in the HT-29 colon cancer cell line is followed by decreased invasiveness and tumorigenicity [14]. In addition it has been shown that furin, another member of the convertase family, is implicated in tumor progression in human head and neck malignancies [15]. Convertase overexpression can also alter the growth behavior and the drug responsiveness in a human breast cancer cell line model [16]. In the past, 7B2 has been implicated with several types of neuroendocrine tumors, such as neuroendocrine bronchial tumors, nonfunctioning pancreatic islet tumors and ACTH-secreting pituitary tumors [17,18], mainly participating at the processing of tumor-secreted active peptides. However its role in tumorigenesis and metastasis, especially in colorectal cancer, remains largely unknown.

Other groups have also recently showed the implication of several members of the convertase family in the carcinogenesis process. Specifically *Siegfried et al *have clearly shown that members of the convertase family process VEGF-C, a known tumorigenic growth factor, in animal models [19]. The same group has demonstrated that convertases are involved in the processing of pro-platelet derived growth factor A, a hallmark of carcinogenesis [20], thus concluding that convertase inhibitors might be used eventually in the treatment of neoplasia.

Our data support the accumulation of active PC1 and PC2 in metastasis, and are in agreement with previous reports [3,16]. The main question although remains whether alterations of PC1, PC2 and 7B2 expression profiles are the cause or consequence of the metastatic phenotype. PC1 and PC2 process pro-neurotensin to its active form, which has been involved in colonic tumorigenesis [21,22], or pro-pancreatic peptide, proGHRH, proglucagon, prosomatostatin, and pro-insulin to insulin [23,24], which are known trophic factors for the gut and possibly involved in tumorigenesis as well. Indeed, recently it was shown that PC1 null mice are dwarfed, thus implicating PC1 in the processing of GHRH and subsequent growth [23]. Therefore change in PC1 and PC2 expression and activation may alter the profiles of secretory proteins, which in turn could increase cell growth potential. These changes could lead to selection of primary colon cancer cells destined to metastasize to the liver or even render the rest of the liver susceptible to future/further metastasis. Indeed, the fact that the PC2 profile observed in unaffected liver is partially different from the one observed in normal liver, supports the hypothesis that the tumor may induce modifications in the rest of the liver. Whether PC1, PC2 and 7B2 are directly implicated in such a model is under investigation, and it would be interesting also to evaluate the profile of these convertases in different also metastatic sites such as in the lung or in the brain.

In conclusion, the present study shows that PC1 and PC2 convertase expression and cleavage are altered in CRC liver metastases. 7B2, whose overexpression in tumor is thought to play a key role in the above processes, could represent a potential diagnostic, prognostic or even therapeutic target.

Furthermore, the metastasis/tumor associated convertase profile is observed only in a fraction of primary colon cancers thus suggesting a potential selection process for tumors that eventually will develop metastasis and may be associated with worse clinical outcome. These observations require prospective validation that is underway.

## Competing interests

The author(s) declare that they have no competing interests.

## Authors' contributions

George N Tzimas: study conception, performed the majority of PCR and immunoblot, wrote the manuscript

Eric Chevet: performed immunoblots and critically revised the manuscript

Sarah Jenna: performed immunoblots and PCR, revised the manuscript

Duc Thang Nguyên: performed part of PCRs

Abdel Majid Khatib: performed immunoblots

Victoria Marcus: evaluated immunohistochemistry

Yi Zhang: performed immunohistochemistry

Michel Chrétien: critically revised the manuscript

Nabil Seidah: provided antibodies and primers, revised the manuscript

Peter. Metrakos: provided specimens and reviewed the manuscript.

## Pre-publication history

The pre-publication history for this paper can be accessed here:
